# An online monitor of the oxidative capacity of aerosols (o-MOCA)

**DOI:** 10.5194/amt-10-633-2017

**Published:** 2017-02-28

**Authors:** Arantzazu Eiguren-Fernandez, Nathan Kreisberg, Susanne Hering

**Affiliations:** Aerosol Dynamics Inc., 935 Grayson St., Berkeley, CA, USA

## Abstract

The capacity of airborne particulate matter to generate reactive oxygen species (ROS) has been correlated with the generation of oxidative stress both in vitro and in vivo. The cellular damage from oxidative stress, and by implication with ROS, is associated with several common diseases, such as asthma and chronic obstructive pulmonary disease (COPD), and some neurological diseases. Yet currently available chemical and in vitro assays to determine the oxidative capacity of ambient particles require large samples, analyses are typically done offline, and the results are not immediate.

Here we report the development of an online monitor of the oxidative capacity of aerosols (o-MOCA) to provide online, time-resolved assessment of the capacity of airborne particles to generate ROS. Our approach combines the Liquid Spot Sampler (LSS), which collects particles directly into small volumes of liquid, and a chemical module optimized for online measurement of the oxidative capacity of aerosol using the dithiothreitol (DTT) assay. The LSS uses a three-stage, laminar-flow water condensation approach to enable the collection of particles as small as 5 nm into liquid. The DTT assay has been improved to allow the online, time-resolved analysis of samples collected with the LSS but could be adapted to other collection methods or offline analysis of liquid extracts.

The o-MOCA was optimized and its performance evaluated using the 9,10-phenanthraquinone (PQ) as a standard redox-active compound. Laboratory testing shows minimum interferences or carryover between consecutive samples, low blanks, and a reproducible, linear response between the DTT consumption rate (nmol min^−1^) and PQ concentration (μM). The calculated limit of detection for o-MOCA was 0.15 nmol min^−1^. The system was validated with a diesel exhaust particle (DEP) extract, previously characterized and used for the development, improvement, and validation of the standard DTT analysis. The DTT consumption rates (nmol min^−1^) obtained with the o-MOCA were within experimental uncertainties of those previously reported for these DEP samples. In ambient air testing, the fully automated o-MOCA was run unattended for 3 days with 3 h time resolution and showed a diurnal and daily variability in the measured consumption rates (nmol min^−1^ m^−3^).

## 1 Introduction

Although there is ample evidence linking exposure to particulate air pollution to adverse health effects, the mechanisms that lead to those effects are not completely understood. A leading hypothesis is that inhaled ambient particulate matter generates reactive oxygen species (ROS), which in turn create cellular damage and induce oxidative stress (Fitzpatrick et al., 2014; Kelly, 2003; Birben et al., 2012; Xia et al., 2006). Oxidative stress is associated with many well-known, widely spread diseases such as Alzheimer’s, atherosclerosis, diabetes, and myocardial infarction (Andersen et al., 2010; Block and Calderon-Garciduenas, 2009; Calderon-Garciduenas et al., 2008; Maritim et al., 2003; Singh and Jialal, 2006; Uttara et al., 2009). The oxidative potential of airborne particles is attributed not only to their chemical composition (organics, trace metals; Eiguren-Fernandez et al., 2010; Ercal et al., 2001; Jomova and Valko, 2011; Chien et al., 2009; Hawley et al., 2014) but also to their physical characteristics (particle size, shape, etc.; Bünger et al., 2000; Chien et al., 2009; de Haar et al., 2006; Wessels et al., 2010). Trace metals, such as copper and iron, can generate ROS via Fenton chemistry, while organics, including polycyclic aromatic hydrocarbons (PAHs) and quinones, generate ROS via metabolic transformations (Chung et al., 2006; Kumagai et al., 2012; Ercal et al., 2001; Valko et al., 2005).

To quantify the oxidative potential of airborne particulate matter, both in vivo and in vitro studies have been conducted. These studies have found correlations between the oxidative potential of airborne particles and the production of biological markers of ROS formation and oxidative stress (Bardet et al., 2014; Hawley et al., 2014; Li et al., 2004; Swanson et al., 2009; Acworth et al., 1999; Kim et al., 2001; Maier et al., 2008; Oberdorster, 2000). Yet the extent of these measurements is limited. Recently, efforts have been made to reduce and replace the use of animal models through cellular and chemical assays. Chemical assays are more rapid and less costly, and they offer a simpler means to screen airborne particulate matter toxicity. There are several assays used to assess airborne particle oxidative potential, each with different sensitivities to the ROS-generating compounds (Hedayat et al., 2014). The dihydroxybenzoate (DHBA) and ascorbic acid (AA) assays, which measures the ability of PM to deplete antioxidants, have been shown to be most sensitive to transition metals (Di Stefano et al., 2009; Janssen et al., 2014). The dithiothreitol (DTT) assay (Cho et al., 2005), which is based on the ability of redox-active compounds associated with particulate matter to catalyze the reduction by DTT of oxygen to superoxide, has been correlated with both organics and transition metals (Sauvain et al., 2015). The 2′,7′-dichlorofluorescin diacetate (DCFH-DA) assay is based on the oxidation of DCFH based on the principle that non-fluorescent fluorescein derivatives will emit fluorescence after being oxidized by hydrogen peroxide (Hung and Wang, 2001; Huang et al., 2016).

Among these methods the dithiothreitol (DTT) assay (Cho et al., 2005) developed to measure the oxidative capacity of airborne particles has become widely adopted (Steenhof et al., 2011; Janssen et al., 2014; Charrier et al., 2015; Godri et al., 2011). The acceptance of this assay is based on several studies that show a high correlation between the DTT assay and more specific biological markers of oxidative stress such as heme oxygenase 1 (HO-1) and inflammatory markers such as interleukin-6 and interleukin-8 (Li et al., 2003; Steenhof et al., 2011; Jiang et al., 2016), and granulocyte macrophage colony-stimulating factor (GM-CSF; Hussain et al., 2009; Uzu et al., 2011). Recent epidemiological studies have also found an association between the DTT-measured oxidative potential and various health end points such as asthma and congestive heart failure (Bates et al., 2015; Fang et al., 2016; Yang et al., 2016). The DTT assay is based on the ability of redox-active compounds associated with particulate matter to catalyze the reduction by DTT of oxygen to superoxide. Currently the DTT assay is done offline; particles collected on filters are incubated with DTT over several periods between 10 and 45 min, and after each time point the reaction between the DTT and the redox-active components is quenched by the addition of 5,5′-dithiobis-2-nitrobenzoic acid (DTNB), forming a chromophore with an absorbance at 412 nm than can be measured to determine the rate of DTT consumption over time. The total oxidative capacity of the particulate matter is expressed as the rate of DTT consumed per unit particle mass (nmol DTT min^−1^ μg^−1^) or per sampled air volume (nmol DTT min^−1^ m^−3^). This approach has the ability to distinguish between the contribution of metals and of organics to the overall oxidative capacity by the selective removal of metal-associated activity before conducting the assay. The addition of a metal chelator to the solution prior to the assay eliminates the metal-associated activity to obtain the oxidative activity due to organics alone. Thus, the contribution of metals to the overall oxidative potential of the sample is obtained from the difference in the DTT activities without and with the chelator (Eiguren-Fernandez et al., 2010, 2015). Although simpler than the in vivo and in vitro methods, the offline DTT assay requires many steps, and the accuracy of the measurement is limited by the integrity of the collected sample since typically long collection periods (24–48 h) and filter extraction processes that follow may alter the chemical and toxicological properties of the particles.

Several online systems developed to monitor the oxidant capacity of airborne particles have been reported previously. King and Weber (2013) reported a method using a mist chamber to capture soluble gases and particles, coupled to automated analysis based on the DCFH assay. Venkatachari and Hopke (2008) and Wang et al. (2011) reported an automated DCFH assay method for measurement of ROS activity for the water-soluble component of ambient particles captured with a Particle into Liquid Sampler. Yet the DCFH assay is not as specific as the DTT assay described above, reporting also activity related to reactive nitrogen compounds. In addition, a study conducted by Chen et al. (2010) showed that photooxidation by the laser light utilized for fluorescence excitation in the DCFH assay can create false-positive results and background values increase with time. Moreover, the horseradish peroxidase (HRP) enzyme is used commonly in this assay to catalyze the generation of OH radicals and to improve the detection of target molecules (ROS). The presence of HRP in the reaction mixture induced a three-fold increase in DCFH oxidation, which can further lead to the overestimation of the measured oxidative potential (Pal et al., 2012.

A more recent online method using the DTT assay is reported by Sammeenoi et al., who coupled their analysis to a Particle into Liquid Sampler (Sameenoi et al., 2012). This sampler uses steam injection to condensationally enlarge the particles and impacts the droplets onto a surface continually washed by the water condensate, thus capturing the water-soluble components associated with the collected particles. As both water-soluble (Fang et al., 2016) and insoluble components (Shinyashiki et al., 2009; Verma et al., 2012; Wang et al., 2013; Li et al., 2015) of airborne particles have been associated with the oxidative potential, it is important to have the ability to measure the contribution of each fraction to the total oxidative potential. This is important as each fraction may have different physiological effects (Delfino et al., 2010).

In this paper, we present a new approach to provide online measurements of the oxidative capacity of airborne particles using the DTT assay and including both insoluble and soluble airborne particle constituents. Our approach uses the Liquid Spot Sampler (LSS) as our particle collector. This system enlarges particles through water condensation at moderate temperatures in a laminar flow and then directs the flow through a set of impaction jets to the surface of a liquid where the droplets are deposited inertially. Through choice of the jet configuration and flow rate, the insoluble particles remain suspended in the capturing liquid, and thus the LSS captures both insoluble and soluble components. Reported here is an online chemical module based on the DTT assay that coupled to the LSS enables in situ, time-resolved characterization of the ability of aerosols to generate ROS.

## 2 Materials and methods

### 2.1 Liquid Spot Sampler

The LSS is the front end of the system – it deposits both soluble and insoluble components of airborne particulate matter into a small volume of liquid. The LSS uses the three-stage water condensational growth technology (Lewis and Hering, 2013; Hering et al., 2014; Eiguren Fernandez et al., 2014) to enlarge submicrometer particles to form micrometer-sized droplets that are subsequently deposited into a water-filled vial. The supersaturation required for condensational growth is created in a laminar flow through a wet-walled tube, the first portion of which is cooled to ~ 5 °C, the second portion is warmed to ~ 37 °C, and the final portion is again cooled to ~ 12 °C. As shown by Lewis and Hering (2013), this approach activates the condensational growth of sub-10 nm particles. In contrast to steam injection condensation methods cited above, the temperature of the majority of the flow remains below 30 °C. Further, at the exit of the condensation region the water vapor content of the droplet laden flow is below saturation at ambient temperature, enabling direct collection into liquid without excessive water condensation from the vapor. The LSS uses three parallel growth tubes, each one running at 1.0 liter per minute (LPM) and delivering condensationally enlarged particles through separate 1.1 mm jets to gently impact the droplets directly onto the surface of water (200 to 500 μL) contained in a small vial. The sample flow rate of the o-MOCA configuration can vary from 1.5 to 4.0 L min^−1^, although liquid collectors with higher flow rates could be used with higher collection volumes (Lednicky et al., 2016; Pan et al., 2016). Uniquely, our approach provides particle growth and collection at moderated temperatures (< 30 °C), reducing possible artifacts and maintaining sample integrity.

The collection efficiency of the LSS was evaluated in our laboratory (Berkeley, CA) using both hydrophilic (sulfate and nitrate) and highly hydrophobic (sebacic acid) particles. Particle collection efficiency was inferred through measurement of particle penetration through the sampler after delivery into the liquid. For submicrometer particle sizes, the aerosol was generated through atomization, mixing 10 : 1 with dry air; charge-neutralized using a soft X-Ray source (TSI model 3087); and size-selected using a high-flow differential mobility analyzer (HF-DMA; Stolzenburg et al., 1998). The DMA was operated with single-pass, 15 L min^−1^ sheath flow comprised of filtered laboratory air (with relative humidity of 30–40 %). Particle concentrations at the inlet to the sampler and in the exiting flow were monitored using a pair of water-based condensation particle counters (TSI Model 3783 upstream and 3787 downstream). These tests were done as a function of particle size (10 to 200 nm) and sampling flow rates (1.5 to 3.5 L min^−1^).

### 2.2 Chemical module

The DTT assay is conducted in the chemical module after sample collection. The main components of the module are two precision syringe pumps (Cavro XLP6000, Tecan); a six-port analytical injection valve (EV-750, Alltech); a homemade heating/shaking system consisting of a aluminum block, a thermoelectric device, and a shaker (VWR), which contains the reaction vial where the sample and the DTT reagent (Sigma Aldrich) mix and react, a “mixing tee” (Upchurch) where the DTT-sample-containing solution and DTNB (Sigma-Aldrich) join and are mixed, and a diode array detector (DAD, Agilent 1100). PEEK and Teflon were selected as the materials for all liquid handling tubing to reduce unwanted chemical reactions.

**Figure 1:**
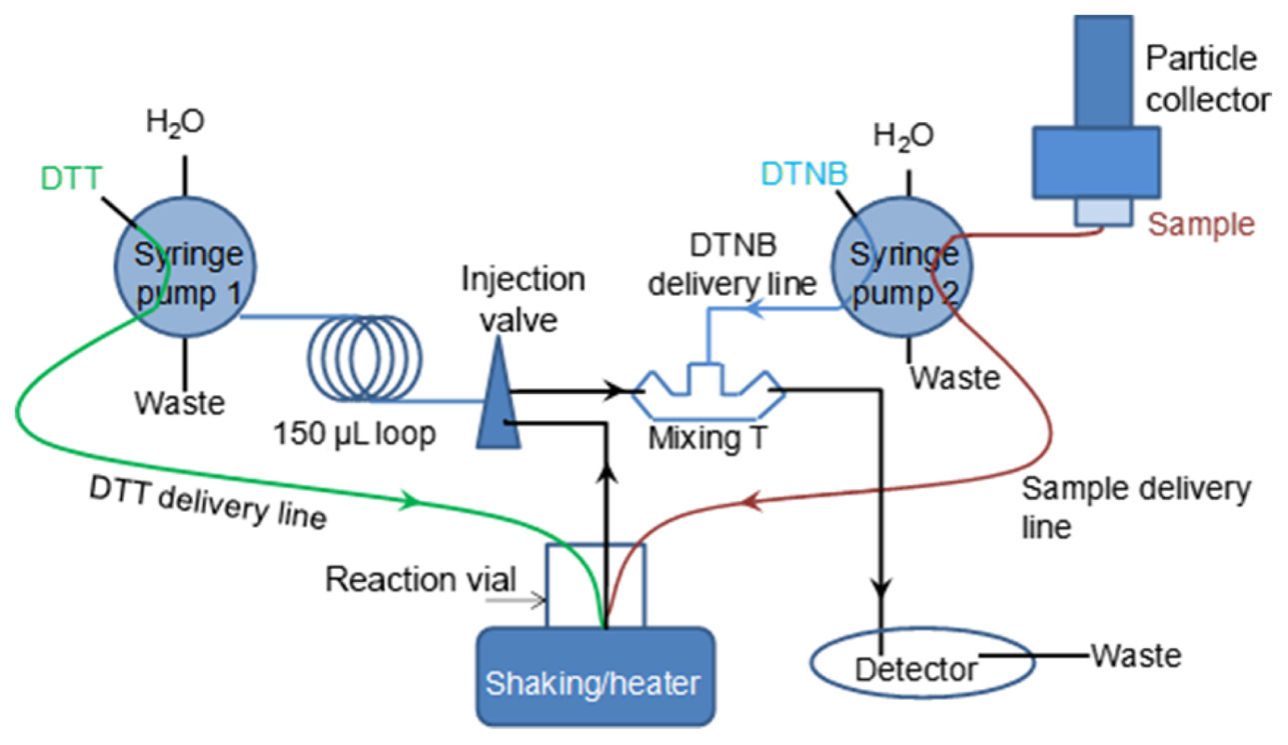
Flow diagram for the online monitor of the oxidative capacity of aerosols (o-MOCA).

The flow diagram of the o-MOCA is shown in Fig. 1. Although our chemical module has similarities to the semiautomated system developed by Fang et al. (2015), the o-MOCA has been improved to couple with a liquid particle collector, to eliminate unnecessary steps in the DTT assay, and to reduce sample cross contamination during online analysis.

As soon as the predetermined sample collection time ends, the whole sample volume is transferred to the reaction vial using syringe pump 2. Once the sample is transferred, the collection vial and lines are rinsed with Chelex-treated water, and 500 μL is added to the vial to initiate a new collection. After these steps, pump 2 starts the continuous delivery of the solution containing DTNB in Chelex water (2 μmol) to the detector. Several minutes later, once the system is equilibrated, 500 μL of DTT solution (100 nmol) in phosphate buffer (0.1 M, pH 7.4, Chelex treated) is added to the reaction vial via syringe pump 1. The solution containing the sample and DTT is incubated at 37 °C with gentle shaking. At the designated times of 1, 3, 5, 7.5, 10, 12.5, and 15 min into the reaction, syringe pump 1 extracts 75 μL of solution from the reaction vial and, through a 150 μL loop, delivers it via the injection valve to the mixing tee, where it combines with the DTNB flow prior to reaching the detector, forming 5-mercapto-2-nitrobenzoic acid that is measured by absorbance at 412 nm. The reaction between the DTT in solution and the DTNB is fast, and since delivery to the detector is quick there is no need for adding stabilizing agents such as trichloroacetic acid or Tris buffer (Charrier and Anastasio, 2012; Charrier et al., 2015; Cho et al., 2005). Removing these extra processing steps, which dilute the sample, results in a more concentrated solution and increased sensitivity. Once the analysis is finished, and prior to delivering the next sample, the reaction vial is rinsed twice with double-distilled, Chelex-treated water. All solution deliveries and rinsing steps are controlled via computer (Labview, National Instruments). A separate analysis routine (Igor, WaveMetrics) is used to post-process the DAD signal data to identify and measure maximum absorbance corresponding to each aliquot injection. In the future, the control and analysis software could be combined into a single program for immediate data reporting.

The possibility of having particle loses during collection and transport of the suspension to the reaction vial has been considered. While not directly measured, losses to collection in the vial and during transport to the reaction vial are expected to be minimal. With our system both insoluble and soluble components or the particles are collected directly into water. Insoluble particles remain suspended in the water collection medium and have little opportunity to interact with vial and tube walls. In addition, suspended particles are close to being neutrally buoyant, rendering inertial forces negligible. A study conducted by Matas et al. (2004) has shown that neutrally buoyant particles are driven away from walls in a laminar tube flow when particle dimensions approach those of the tube diameter. Jochem et al. (2016) found that transport of particles in microfluidic devices and losses in comparable PEEK tubing were negligible. Based on these considerations and studies, we assume there is little opportunity for water-encapsulated particles to be lost to surfaces during both collection into water and transport.

### 2.3 System optimization and validation

System optimization was done using the 9,10-phenanthraquinone (PQ), a very active redox compound commonly used for evaluating the DTT assay. Its redox-active behavior has been well characterized, and several studies have shown a linear relation (first-order reaction) between the rate of DTT loss and the PQ concentration in solution (Charrier and Anastasio, 2012; Fang et al., 2015). The carryover and cross contamination between injections were examined by running consecutive samples containing different amounts of the PQ standard. To evaluate if higher activity samples had an effect over subsequent samples of lower activity, we measured the DTT loss rate of blanks followed by high PQ concentrations (0.25 μM) followed by low PQ concentrations (0.05 μM).

An important parameter to consider while developing the online system to measure time-resolved oxidative capacity of airborne particles is the sensitivity of the assay. A previous study conducted by Sameenoi et al. (2012) showed that increasing the initial DTT added to solution resulted in a reduction of the sensitivity of the assay. Although their method of analysis was different to ours, we considered this parameter when optimizing our online assay. The effect of the initial amount of DTT in solution on the observed consumption rate was evaluated through systematic increases in the mass of DTT added to the reaction vial (50, 100, and 140 nmol) at a fixed PQ concentration (0.25 μM).

Our long-term goal is to develop a field-deployable o-MOCA that can run unattended for extended periods of time. However, as the DTT is a chemical assay, and both the DTT and the DTNB are known to undergo degradation when exposed to light and high temperatures, optimal conditions for maintaining the integrity of these solutions for long sampling periods were determined. Photodecomposition was eliminated by keeping the reagent containing reservoirs in the dark (wrapped in aluminum foil). The effect of ambient temperature on the stability of the reagent was evaluated in two different ways: (i) by measuring the consumption rate of a blank solution for consecutive runs over a 7 h period and (ii) by measuring and comparing daily the absorbance of the DTT–DTNB mixture at *t* = 0 for solutions kept in the refrigerator (4°C).

Once the chemical module was optimized, the system was run continuously for several hours to measure the DTT consumption rate and linearity for different concentrations of PQ. Further validation of the online method was conducted using a diesel exhaust particle extract (DEP; courtesy of Arthur Cho and Debra Schmitz, University of California Los Angeles (UCLA)). This DEP was used to develop the original DTT assay and later as a control for the improved method (Cho et al., 2005). It has been used as a positive control when measuring the activity of ambient particulate matter in all the studies conducted by Arthur Cho’s group. It is also used by Ning Li (Michigan State University) as a positive control for in vitro assays when measuring biomarkers of oxidative stress.

### 2.4 Online measurement of the oxidative capacity of ambient aerosols

The fully automated o-MOCA was tested for continuous online measurement of ambient aerosols in our laboratory (ADI, Berkeley, CA). Our prototype o-MOCA ran unattended, without operator interaction, over a 3-day period. Ambient particulate matter samples collected at 3 L min^−1^ for 3 h, and the oxidative potential of the sample was measured at the end of each 3 h collection period. While the DTT assay was conducted, a new sample was collected. The particle oxidative capacity of the ambient particles is reported as the rate of DTT consumption per square meter (m^−3^) of air collected (nmol DTT min^−1^ m^−3^).

## 3 Results

### 3.1 Collection efficiency of the Liquid Spot Sampler

**Figure 2:**
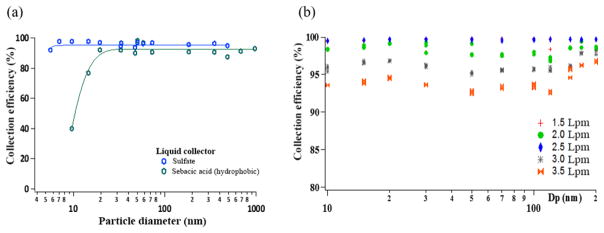
Collection efficiency for laboratory-generated salt and sebacic acid aerosols with the Liquid Spot Sampler: **(a)** with particle size and **(b)** with sampling flow rate for salts.

The collection efficiency for mixed salt particle size and sampling flows, and highly hydrophobic sebacic acid are shown in Fig. 2. Hydrophobic particles showed collection efficiencies of 80 % for 15 nm particles, increasing to > 90 % for larger particle sizes (Fig. 2a). For hydrophilic salts collection efficiencies > 90 % were obtained for all particle sizes and flows (Fig. 2a and b). Higher collection efficiencies (> 97 %) were observed for sampling flow rates varying between 1.5 and 2.5 L min^−1^; the efficiency decreased slightly as the sampling flow rate increased to 3.0 (95 %) and 3.5 L min^−1^ (93 %). A sampling flow rate of 3.0 L min^−1^ was selected to run the Liquid Spot Sampler to maximize the air sample volume collected without compromising the high collection efficiency over a wide range of particle sizes.

During these experiments, the volume of the collecting liquid was adjusted according to the sampling flow. For lower flow rates, smaller volumes could be used (down to 200 μL) without observing changes in the collection efficiency. For the higher flow rates, the minimum collection volume was increased to 500 μL. For a smaller volume, the incoming jets disrupted the liquid surface, depositing the particles onto the dry surface of the bottom of the collection vial. In this case, although the collection efficiency was not greatly affected, resuspension of the deposited particles was required. Gentle agitation was enough to remove particles from the surface and into the liquid. Direct deposition of the sample into water is preferred to ensure non-soluble particles are entrained. For the 3.0 L min^−1^ sampling flow rate used with the o-MOCA, 500 μL was selected as the volume for optimum liquid collection.

### 3.2 System optimization and validation

#### 3.2.1 Equivalence of DAD to benchtop UV–Vis detector

In our online system, we use a continuous-flow DAD instead of a batch fixed-wavelength (412 nm) UV–Vis detector. Our first step was to evaluate the equivalence between the readings of the benchtop UV–Vis detector (Spectronic 20D, Milton-Roy) and the online method with the DAD detector for the same sample. We ran the standard offline DTT assay (Cho et al., 2005) using PQ as our standard compound, and the absorbance of the same solution after 0, 5, 10, and 20 min reaction was measured with both detectors. Good linear relation was observed between the detectors (*r*^2^ = 0.99). As the analog output signal of the DAD is not recorded as arbitrary units (AU) but as volts, the absorbance signal measured directly by the DAD and the output voltage recorded by the system were also compared. Although the range of the processed voltage output was lower than the signal displayed (AU), the correlation between both measurements was good, indicating that the analog output can be used to accurately determine the absorbance of the solution.

#### 3.2.2 Injection integrity, cross contamination, and carryover

**Figure 3:**
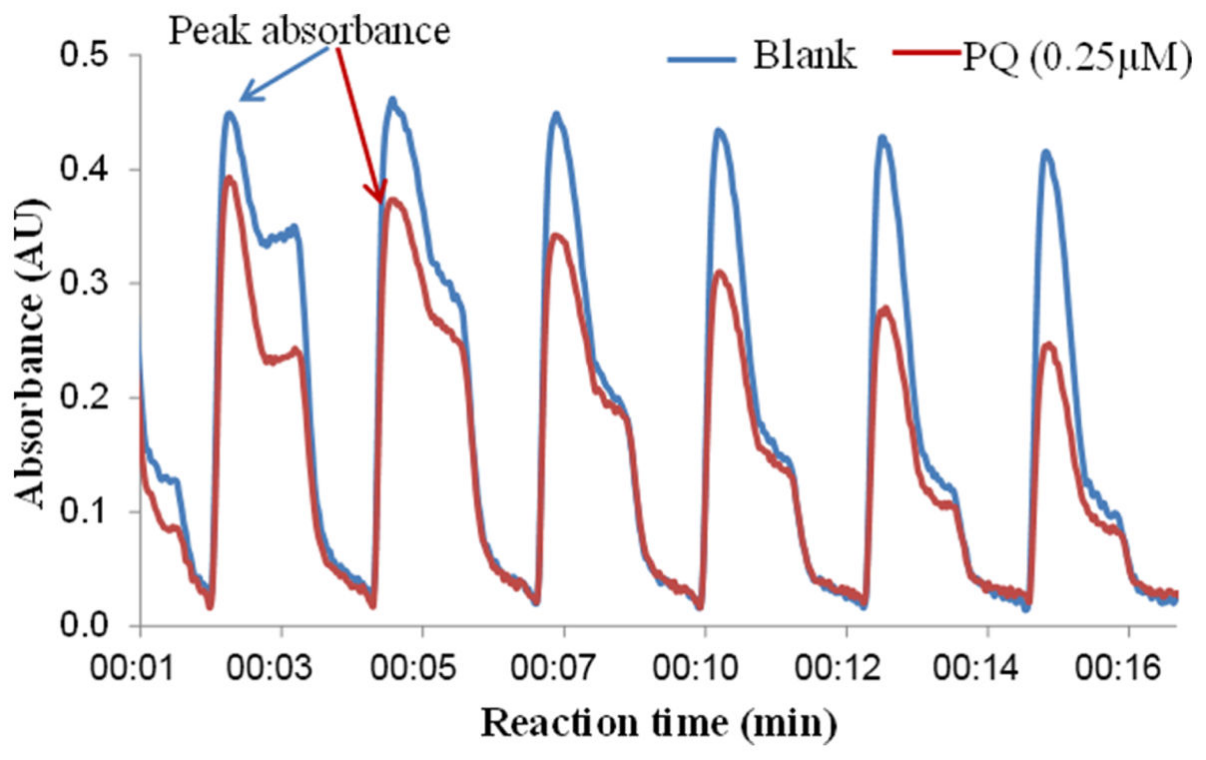
Peak absorbance for water blanks and PQ (0.25 μM) after rinsing injection with water.

As in many liquid-based online systems, the potential for carryover from injection to injection and sample to sample is of concern. Although the internal volume of the lines has been minimized, the dead volume of the syringe pumps could lead to cross contamination. To eliminate interferences and cross contamination between consecutive injections, we added a 150 μL loop and a six-port injector valve between the reaction vial and syringe pump. The loop line, filled with water, prevents the reaction solution from reaching the syringe pump, eliminating undesired mixing. We also added a step to rinse the delivery lines between injections by injecting 75 μL of water following the 75 μL of reaction solution using pump 1. The resulting peaks after implementation of the loop and rinsing step for a blank and PQ solutions are shown in Fig. 3. No interferences were observed for the sample peak height, suggesting that the rinsing step with water injection reduces cross contamination without interfering with the sample signal.

After this improvement, we tested the new configuration for possible carryover using different standard amounts. The DTT consumption rate (nmol min^−1^) obtained for each sample is shown in Table 1. The difference obtained for the duplicates suggests that, although small (< 10 %), there may be some carryover effect when considerable changes in concentration (e.g., a factor of 5 as in this test) occur between consecutive samples. This small effect could be further reduced with design improvements.

#### 3.2.3 Optimization of the initial DTT mass in solution

**Figure 4:**
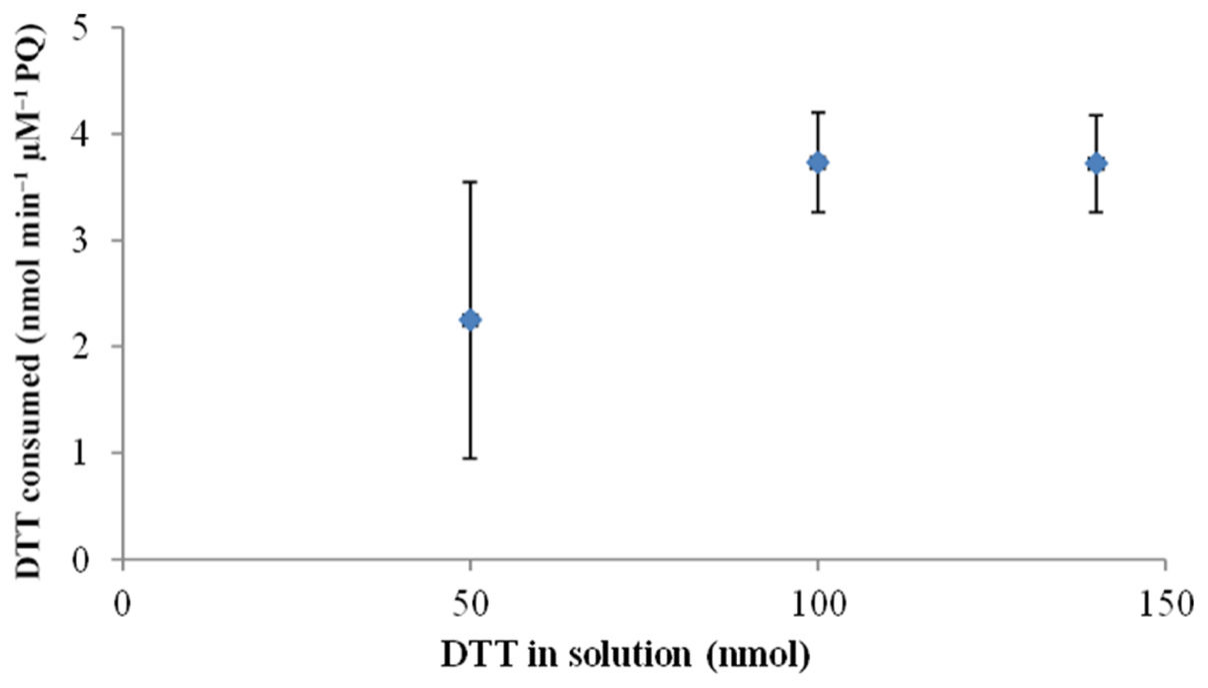
DTT consumption rate (nmol min^−1^ μM^−1^ PQ) for different initial DTT concentrations in solution (PQ concentrations of 0.25 μM).

Figure 4 shows the average consumption rates (±SD) for each initial DTT concentration. Results showed that at low initial amounts of DTT in the reaction solution the consumption rate was slightly lower than expected, with higher variability in the calculated rate. For initial concentrations of 100 and 140 nmol, the consumption rate was very similar, with lower variability for consecutive samples. Based on these results, the addition of 100 nmol was selected for further studies. A similar study was conducted to evaluate the effect of DTNB concentration on the intensity of the solution after reaction. Two different DTNB concentrations, 1 and 2 μmol, were used, and the results (not reported here) indicated no difference between concentrations.

#### 3.2.4 Stability and integrity of the reagent for long-term field deployment and sampling

When reagents were kept at ambient conditions, a slow decrease in the absorbance peak of the DTT–DTNB mixture, estimated for *t* = 0, was observed over time (*n* = 7); however, the consumption rate for all samples (blanks and PQ) remained constant and similar to those obtained in previous and subsequent runs. For solutions kept in iced water, the estimated absorbance at *t* = 0 was very similar over the entire period of analysis (*n* = 7), with again no changes in the consumption rates. In the longer study, DTT and DTNB solutions were kept in the refrigerator (4 °C), and aliquots were taken out at the beginning of each day for 7 consecutive days. The measured absorbance intensity showed a small but not significant decrease over the 7-day period, which did not affect the measured consumption rate. Based on these results, we improved the system by adding a small cooling bath with a recirculating chiller (VWR) to maintain DTT and DTNB solutions at ~ 6 °C for further studies.

#### 3.2.5 System performance for continuous runs using PQ standard

**Figure 5:**
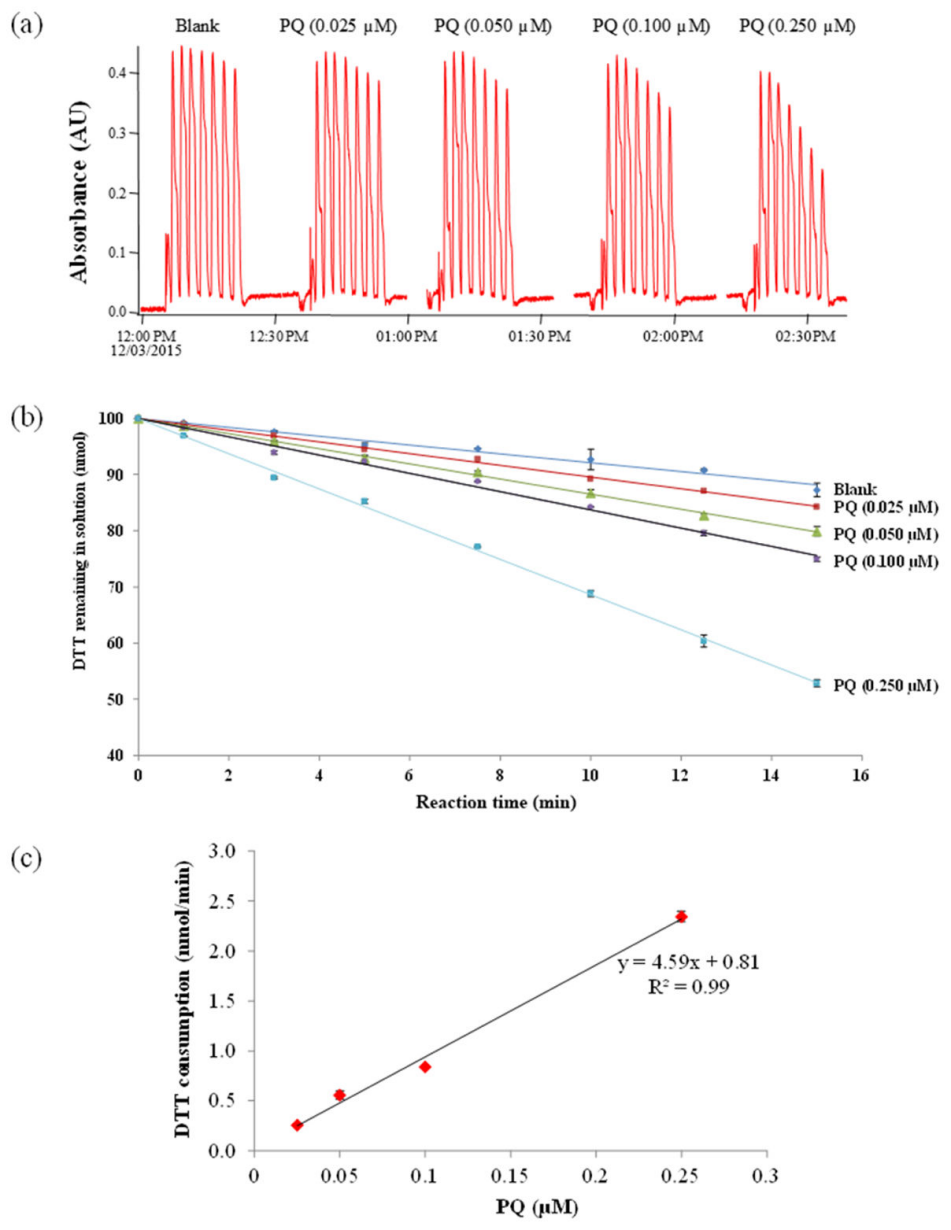
Laboratory evaluation and performance of the automated system for different PQ solutions and blanks: **(a)** raw intensity signal (AU); **(b)** DTT remaining in solution at each reaction time (nmol); **(c)** correlation between DTT consumption rate (nmol min^−1^) and PQ concentration (μM).

Figure 5 illustrates how the oxidative capacity of the samples is obtained with the o-MOCA system. Shown are data obtained for duplicate blanks and PQ samples. Figure 5a shows the raw absorbance data for each of four different quantities of PQ after addition of 500 μL of standard solution for the final concentration in the reaction vial, after adding 500 μL of DTT, of 0.025, 0.05, 0.1, and 0.25 μM, plus a blank. Each group of peaks shows the decay in DTT concentration over time due to consumption by the PQ. Figure 5b shows the transformed data, calculated as the fraction of DTT remaining in solution (average ±SD) versus the reaction times. The amount remaining is obtained by normalizing the absorbance values by the initial absorbance that is inferred by the zero intercept in a linear fit (corresponding to 100 nmol) to the absorbance values obtained at each reaction time (excluding the first two peaks, corresponding to 1 and 3 min). For all samples a good linear correlation between the fraction of DTT remaining and reaction time was observed, with a small standard deviation for duplicates. The rate of DTT loss was determined for each PQ concentration from the slope and zero intercept of the linear regression for the measured absorbance, as shown in Fig. 5b. Multiplying the blank subtracted values by the DTT added to initiate the reaction gives the consumption rate for each PQ concentration in solution. The average rate of DTT consumption (±SD) obtained from all runs is 9.8 ± 1.2 nmol DTT min μM^−1^ PQ.

Figure 5c presents the regression equation for DTT consumption for this range of PQ concentrations. As expected, a linear correlation is obtained with near-zero intercept, indicating a good system response to this standard compound. The small standard deviation and consistency of these data which span an order-of-magnitude variation in the PQ concentrations indicate that the online DTT method works properly.

The limit of detection (LOD) of the system, defined as 5 times the standard deviation of the slope obtained for our blanks (*n* = 12 over different days), is 0.15 (nmol min^−1^). This value is lower than previous limits of detection reported in the literature for the DTT assay (Charrier and Anastasio, 2012; Fang et al., 2015). If we express the limit of detection as equivalents of PQ mass in solution using the regression obtained in Fig. 2c, our LOD is close to the 0.025 μM PQ (25 pmol) in the reaction solution that we have been using as our lowest test concentration. These results validate our online DTT method, which eliminates the need for quenching agents through rapid assay, proving a method that is both simpler and more sensitive yet that retains the robustness of the original method.

### 3.3 Validation of the online DTT assay by comparison with UCLA standard method

To validate our improved method, we ran the online DDT assay using DEP extract (in DMSO) obtained from UCLA (courtesy of Arthur Cho and Debra Schmitz). We measured the DTT consumption rate for three different initial amounts of DEP (*n* = 2) in the incubation solution as done at UCLA (data obtained from Emma DiStefano). Table 2 shows the DTT consumption rate obtained using our o-MOCA and the ones obtained by the UCLA group. Similar linear regression curves were obtained for the DTT consumption rate (nmol min^−1^) as a function of DEP mass for both methods, with *y* = 0.031 + 0.929 for the o-MOCA and *y* = 0.036 + 0.591 for UCLA.

These results indicate that our improved assay is comparable to the original DTT method and that the reported consumption rates will be directly comparable to laboratory-based assays with equal accuracy.

### 3.4 Online measurement of the oxidative capacity of ambient aerosols

The interface of the chemical module with the Liquid Spot Sampler was done connecting a port from one of the syringe pumps to the sample collection vial. The line is connected to the bottom of the collection vial to remove the entire particle-containing suspension. The liquid suspension, containing both water-soluble and water-insoluble material, is then delivered to the reaction vial where the reaction with DTT takes place. After sample delivery, the sample collection vial is rinsed twice with double-distilled water (DDW) (Chelex) and filled again for the next sample collection. All these steps are conducted automatically. After some preliminary testing, the original collection vial was modified slightly to provide more complete sample transfer into the chemical module. We also found that suspension transfer was aided by turning the sampling flow off during the transfer and rinsing steps. A solenoid valve under software control was added to enable automatic switching off/on of the sample flow. The improved and optimized o-MOCA interface was initially evaluated using different standard solutions of PQ (as shown in Fig. 5). Finally, to accommodate the analysis of an air sample containing insoluble as well as soluble particulate matter, we added a stainless-steel frit (Upchurch) in the line through which the sample extracted from the reaction vial is delivered to the injection valve. This way, particles are physically present in the suspension while conducting the reaction but do not reach the injection valve, the mixing tee, or the detector, avoiding interferences and clogging of the lines. No differences were observed in DTT consumption rates for PQ solution with and without the frit.

The fully automated o-MOCA was tested for continuous online measurement of ambient aerosols in our laboratory. Our prototype o-MOCA ran unattended over a 3-day period. Ambient PM_2.5_ samples were collected for 3 h, and the oxidative capacity of the sample was measured at the end of the collection. While the DTT assay was conducted, a new sample was collected. It is important to note that DTT and DTNB stability, measured as the absorbance of the mixture at *t* = 0, was good, with a small but not considerable change between injections over the 3-day period.

**Figure 6:**
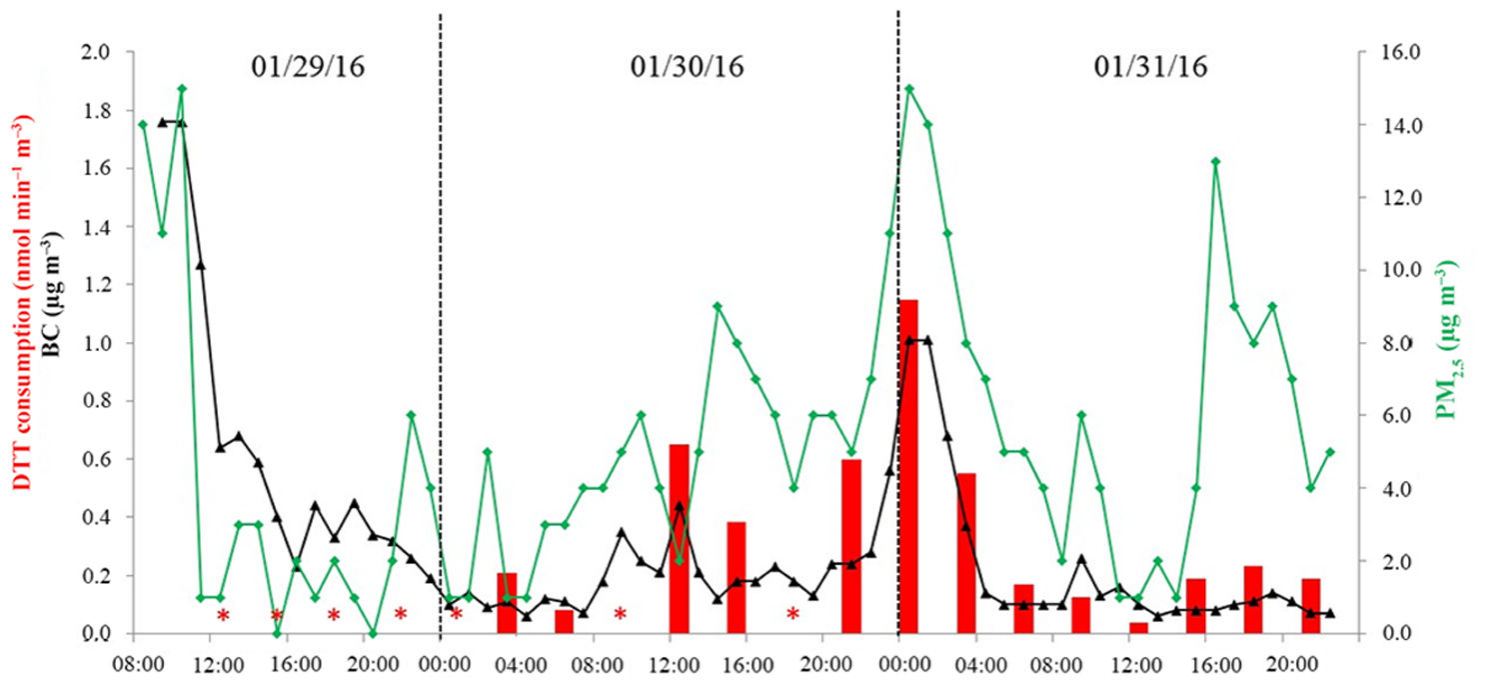
DTT consumption rate (nmol min^−1^ m^−3^) for 3 h samples collected and analyzed using o-MOCA for 3 consecutive days at Berkeley, CA; PM_2.5_ and black carbon (μg m^−3^) data obtained from nearby monitoring station (AQMD); * < LOD.

Figure 6 shows the oxidative capacity of the consecutive 3 h samples measured as the consumption rate of DTT nmol min^−1^ m^−3^. At the beginning of the test the consumption rate was below our limit of detection. During these first days the weather was not favorable with considerable rain. The rain lasted from the beginning of the sampling to midnight on Friday (Weather Underground historical data), which is consistent with the < LOD consumption rate measured during this period. After the rain, changes in the oxidative capacity were observed over time, with considerable diurnal and daily variability. We compared the consumption rate measured for our 3 h samples with hourly concentrations of ambient PM_2.5_ and black carbon (BC) measured at the Bay Area Air Quality Management District (AQMD) monitoring station closest to our location (1.5 km; Fig. 6). The oxidative capacity measured for ambient particles using the o-MOCA followed a similar trend to ambient PM_2.5_ and black carbon concentrations. To better assess the validity of the results obtained with the o-MOCA, we determined the correlation between the DTT activity measured with the o-MOCA and PM_2.5_ mass and BC concentrations measured at the nearby AQMD station. PM_2.5_ and BC hourly data were averaged for the 3 h period of collection of the o-MOCA. Good correlations with both PM_2.5_ mass (*r*^2^ = 0.60) and BC (*r*^2^ = 0.72) were obtained. Previous studies have also shown good correlation between DTT and mass and BC (Janssen et al., 2014; Hu et al., 2008).

The measured activity for airborne particles collected in Berkeley, reported as the DTT consumption rate (nmol min^−1^ m^−3^), is within the range of activities reported by other studies for ambient particulate matter (Cho et al., 2005; Eiguren-Fernandez et al., 2010; Verma et al., 2009). Our results illustrate that the oxidative capacity of ambient particulate matter changes rapidly, and thus good time resolution is important to characterize and determine sources for the oxidative potential of ambient aerosols. Average consumption rates obtained from long sampling periods may fail to measure and report peak oxidative activities.

## 4 Conclusions

We present a method for the online, time-resolved measurement of the oxidative potential of ambient particulate matter. The o-MOCA is a prototype system which allows automated collection and analysis of ambient particles by combining a Liquid Spot Sampler with a newly developed online chemical module. The LSS collects both soluble and insoluble components of airborne particles as concentrated samples of water suspensions. The chemical module has been developed and optimized to streamline the DTT analysis of the oxidative potential of the sample. Laboratory-based tests using the 9,10-phenanthraquinone and DEP extracts as the redox-active compounds show that the newly developed chemical module for streamlined DTT assay reports similar responses to the standard benchtop DTT assay. The o-MOCA was run continuously and unattended for 3 days in our laboratory, collecting 3 h samples of ambient PM_2.5_. Results provided diurnal and temporal variation of the oxidative potential of ambient particulate matter in Berkeley and correlated well with nearby PM_2.5_ mass and BC concentrations. The results presented here are based on laboratory tests for a prototype o-MOCA. Further advancements will be required for extended field operation. The stability of the reagents for periods longer than 7 days has not been tested. To date, the lifespan of the frit before excessive back pressure develops has not been evaluated. In the configuration reported here, the o-MOCA only reports the total oxidative potential (metals and organic contribution) of the collected sample. Future efforts will center on making the o-MOCA more suitable for field deployment by incorporating built-in absorbance measurement and adding peak analysis software for automated oxidative potential reporting. Additional testing envisioned includes comparisons with parallel filter collection and exploring correlations between the oxidative potential of the ambient particles measured by the o-MOCA and their ROS generation measured using in vitro studies. Improving the DTT assay and chemical module to provide both metal and organic contributions is also anticipated.

## 5 Data availability

All the data presented in this study are available from the authors upon request.

## Figures and Tables

**Table 1:** DTT consumption rate (nmol min^−1^) for consecutive samples of different activities.

	Consumption rate (DTT nmol min^−1^)		
	BLK	PQ (0.25 μM)	PQ (0.05 μM)
Injection #1	0.91	2.93	1.46
Injection #2	1.09	3.22	1.31

**Table 2:** Comparison of consumption rates (nmol min μg^−1^ DEP) for different DEP amounts obtained at UCLA with the standard DTT assay and o-MOCA.

DTT consumption rate (nmol min μg^−1^ DEP)	DEP (μg)		
	2.5	5	10
UCLA	0.07	0.072	0.037
o-MOCA	0.068 ± 0.002	0.054 ± 0.003	0.032 ± 0.007

## References

[R1] Acworth IN, Bogdanov MB, McCabe DR, Beal MF (1999). Estimation of hydroxyl free radical levels in vivo based on liquid chromatography with electrochemical detection, Method. Enzymol.

[R2] Andersen ZJ, Olsen TS, Andersen KK, Loft S, Ketzel M, Raaschou-Nielsen O (2010). Association between short-term exposure to ultrafine particles and hospital admissions for stroke in Copenhagen, Denmark. Eur Heart J.

[R3] Bardet G, Achard S, Loret T, Desauziers V, Momas I, Seta N (2014). A model of human nasal epithelial cells adapted for direct and repeated exposure to airborne pollutants. Toxicol Lett.

[R4] Bates JT, Weber RJ, Abrams J, Verma V, Fang T, Klein M, Strickland MJ, Sarnat SE, Chang HH, Mulholland JA, Tolbert PE, Russell AG (2015). Reactive Oxygen Species Generation Linked to Sources of Atmospheric Particulate Matter and Cardiorespiratory Effects. Environ Sci Technol.

[R5] Birben E, Sahiner UM, Sackesen C, Erzurum S, Kalayci O (2012). Oxidative stress and antioxidant defense. The World Allergy Organization journal.

[R6] Block ML, Calderon-Garciduenas L (2009). Air pollution: mechanisms of neuroinflammation and CNS disease. Trends Neurosci.

[R7] Bünger J, Krahl J, Baum K, Schröder O, Müller M, Westphal G, Ruhnau P, Schulz TG, Hallier E (2000). Cytotoxic and mutagenic effects, particle size and concentration analysis of diesel engine emissions using biodiesel and petrol diesel as fuel, Arch. Toxicol.

[R8] Calderon-Garciduenas L, Solt AC, Henriquez-Roldan C, Torres-Jardon R, Nuse B, Herritt L, Villarreal-Calderon R, Osnaya N, Stone I, Garcia R, Brooks DM, Gonzalez-Maciel A, Reynoso-Robles R, Delgado-Chavez R, Reed W (2008). Long-term Air Pollution Exposure Is Associated with Neuroinflammation, an Altered Innate Immune Response, Disruption of the Blood-Brain Barrier, Ultrafine Particulate Deposition, and Accumulation of Amyloid beta-42 and alpha-Synuclein in Children and Young Adults, Toxicol. Pathol.

[R9] Charrier JG, Anastasio C (2012). On dithiothreitol (DTT) as a measure of oxidative potential for ambient particles: evidence for the importance of soluble transition metals. Atmos Chem Phys.

[R10] Charrier JG, Richards-Henderson NK, Bein KJ, McFall AS, Wexler AS, Anastasio C (2015). Oxidant production from source-oriented particulate matter – Part 1: Oxidative potential using the dithiothreitol (DTT) assay. Atmos Chem Phys.

[R11] Chen X, Zhong Z, Xu Z, Chen L, Wang Y (2010). 2′,7′-Dichlorodihydrofluorescein as a fluorescent probe for reactive oxygen species measurement: Forty years of application and controversy. Free Radic Res.

[R12] Chien SM, Huang YJ, Chuang SC, Yand HS (2009). Effects of Biodiesel Blending on Particulate and Polycyclic Aromatic Hydrocarbon Emissions in Nano/Ultrafine/Fine/Coarse Ranges from Diesel Engine, Aerosol Air Qual. Res.

[R13] Cho AK, Sioutas C, Miguel AH, Kumagai Y, Schmitz DA, Singh M, Eiguren-Fernandez A, Froines JR (2005). Redox activity of airborne particulate matter at different sites in the Los Angeles Basin. Environ Res.

[R14] Chung MY, Lazaro RA, Lim D, Jackson J, Lyon J, Rendulic D, Hasson AS (2006). Aerosol-borne quinones and reactive oxygen species generation by particulate matter extracts. Environ Sci Technol.

[R15] de Haar C, Hassing I, Bol M, Bleumink R, Pieters R (2006). Ultrafine but not fine particulate matter causes airway inflammation and allergic airway sensitization to co-administered antigen in mice. Clin Exp Allergy.

[R16] Delfino RJ, Staimer N, Tjoa T, Arhami M, Polidori A, Gillen DL, George SC, Shafer MM, Schauer JJ, Sioutas C (2010). Associations of primary and secondary organic aerosols with airway and systemic inflammation in an elderly panel cohort. Epidemiology (Cambridge, Mass).

[R17] Di Stefano E, Eiguren-Fernandez A, Delfino RJ, Sioutas C, Froines JR, Cho AK (2009). Determination of metal-based hydroxyl radical generating capacity of ambient and diesel exhaust particles. Inhalation Toxicology.

[R18] Eiguren-Fernandez A, Shinyashiki M, Schmitz DA, DiStefano E, Hinds W, Kumagai Y, Cho AK, Froines JR (2010). Redox and electrophilic properties of vapor- and particle-phase components of ambient aerosols. Environ Res.

[R19] Eiguren-Fernandez A, Di Stefano E, Schmitz DA, Guarieiro ALN, Salinas EM, Nasser E, Froines JR, Cho AK (2015). Chemical reactivities of ambient air samples in three Southern California communities. J Air Waste Manage.

[R20] Eiguren Fernandez A, Lewis GS, Hering SV (2014). Design and Laboratory Evaluation of a Sequential Spot Sampler for Time-Resolved Measurement of Airborne Particle Composition. Aerosol Sci Technol.

[R21] Ercal N, Gurer-Orhan H, Aykin-Burns N (2001). Toxic metals and oxidative stress part I: mechanisms involved in metal-induced oxidative damage, Curr. Top Med Chem.

[R22] Fang T, Verma V, Guo H, King LE, Edgerton ES, Weber RJ (2015). A semi-automated system for quantifying the oxidative potential of ambient particles in aqueous extracts using the dithiothreitol (DTT) assay: results from the Southeastern Center for Air Pollution and Epidemiology (SCAPE). Atmos Meas Tech.

[R23] Fang T, Verma V, Bates JT, Abrams J, Klein M, Strickland MJ, Sarnat SE, Chang HH, Mulholland JA, Tolbert PE, Russell AG, Weber RJ (2016). Oxidative potential of ambient water-soluble PM_2.5_ in the southeastern United States: contrasts in sources and health associations between ascorbic acid (AA) and dithiothreitol (DTT) assays. Atmos Chem Phys.

[R24] Fitzpatrick AM, Park Y, Brown LAS, Jones DP (2014). Children with severe asthma have unique oxidative stress-associated metabolomic profiles. J Allergy Clin Immun.

[R25] Godri KJ, Harrison RM, Evans T, Baker T, Dunster C, Mudway IS, Kelly FJ (2011). Increased Oxidative Burden Associated with Traffic Component of Ambient Particulate Matter at Roadside and Urban Background Schools Sites in London. PloS one.

[R26] Hawley B, L’Orange C, Olsen DB, Marchese AJ, Volckens J (2014). Oxidative Stress and Aromatic Hydrocarbon Response of Human Bronchial Epithelial Cells Exposed to Petro- or Biodiesel Exhaust Treated with a Diesel Particulate Filter. Toxicol Sci.

[R27] Hering S, Lewis G, Spielman S (2014). Moderated, Water-Based Condensational Particle Growth in a Laminar Flow, Aerosol Sci. Technol.

[R28] Hedayat F, Stevanovic S, Miljevic B, Bottle S, Ristovski Z (2014). Review-evaluating the molecular assays for measuring the oxidative potential of particulate matter. Chemical Industry and Chemical Engineering Quarterly.

[R29] Hu S, Polidori A, Arhami M, Shafer MM, Schauer JJ, Cho A, Sioutas C (2008). Redox activity and chemical speciation of size fractioned PM in the communities of the Los Angeles-Long Beach harbor. Atmos Chem Phys.

[R30] Huang W, Zhang Y, Zhang Y, Fang D, Schauer JJ (2016). Optimization of the Measurement of Particle-Bound Reactive Oxygen Species with 2′,7′-dichlorofluorescin (DCFH). Water Air Soil Pollut.

[R31] Hussain S, Boland S, Baeza-Squiban A, Hamel R, Thomassen LC, Martens JA, Billon-Galland MA, Fleury-Feith J, Moisan F, Pairon JC, Marano F (2009). Oxidative stress and proinflammatory effects of carbon black and titanium dioxide nanoparticles: role of particle surface area and internalized amount. Toxicology.

[R32] Janssen NAH, Yang A, Strak M, Steenhof M, Hellack B, Gerlofs-Nijland ME, Kuhlbusch T, Kelly F, Harrison R, Brunekreef B, Hoek G, Cassee F (2014). Oxidative potential of particulate matter collected at sites with different source characteristics. Sci Total Environ.

[R33] Jiang H, Jang M, Sabo-Attwood T, Robinson SE (2016). Oxidative potential of secondary organic aerosols produced from photooxidation of different hydrocarbons using outdoor chamber under ambient sunlight. Atmos Environ.

[R34] Jochem A-R, Ankah GN, Meyer L-A, Elsenberg S, Johann C, Kraus T (2016). Colloidal Mechanisms of Gold Nanoparticle Loss in Asymmetric Flow Field-Flow Fractionation. Anal Chem.

[R35] Jomova K, Valko M (2011). Advances in metal-induced oxidative stress and human disease. Toxicology.

[R36] Kelly FJ (2003). Oxidative stress: its role in air pollution and adverse health effects, Occup. Environ Med.

[R37] Kim S, Jaques PA, Chang M, Barone T, Friedlander SK, Sioutas C (2001). Versatile aerosol concentration enrichment system (VACES) for simultaneous in vivo and in vitro evaluation of toxic effects of ultrafine, fine and coarse ambient particles Part II: Field evaluation. J Aerosol Sci.

[R38] King LE, Weber RJ (2013). Development and testing of an online method to measure ambient fine particulate reactive oxygen species (ROS) based on the 2′,7′-dichlorofluorescin (DCFH) assay. Atmos Meas Tech.

[R39] Kumagai Y, Shinkai Y, Miura T, Cho AK (2012). The chemical biology of naphthoquinones and its environmental implications. Annu Rev Pharmacol.

[R40] Lednicky J, Pan M, Loeb J, Hsieh H, Eiguren-Fernandez A, Hering S, Fan ZH, Wu C-Y (2016). Highly efficient collection of infectious pandemic influenza H1N1 virus (2009) through laminar-flow water based condensation. Aerosol Sci Technol.

[R41] Lewis GS, Hering SV (2013). Minimizing Concentration Effects in Water-Based, Laminar-Flow Condensation Particle Counters. Aerosol Sci Technol.

[R42] Li N, Hao M, Phalen RF, Hinds WC, Nel AE (2003). Particulate air pollutants and asthma: A paradigm for the role of oxidative stress in PM-induced adverse health effects, Cl. Immunol.

[R43] Li N, Alam J, Venkatesan MI, Eiguren-Fernandez A, Schmitz D, Di Stefano E, Slaughter N, Killeen E, Wang X, Huang A, Wang M, Miguel AH, Cho A, Sioutas C, Nel AE (2004). Nrf2 Is a Key Transcription Factor That Regulates Antioxidant Defense in Macrophages and Epithelial Cells: Protecting Against the Proinflammatory and Oxidizing Effects of Diesel Exhaust Chemicals. J Immunol.

[R44] Li Q, Shang J, Liu J, Xu W, Feng X, Li R, Zhu T (2015). Physicochemical characteristics, oxidative capacities and cytotoxicities of sulfate-coated 1,4-NQ-coated and ozone-aged black carbon particles. Atmos Res.

[R45] Maier KL, Alessandrini F, Beck-Speier I, Josef Hofer TP, Diabaté S, Bitterle E, Stöger T, Jakob T, Behrendt H, Horsch M, Beckers J, Ziesenis A, Hültner L, Frankenberger M, Krauss-Etschmann S, Schulz H (2008). Health Effects of Ambient Particulate Matter – Biological Mechanisms and Inflammatory Responses to In Vitro and In Vivo Particle Exposures. Inhal Toxicol.

[R46] Maritim AC, Sanders RA, Watkins JB (2003). Diabetes oxidative stress, and antioxidants: a review. J Biochem Mol Toxicol.

[R47] Matas J-P, Morris JF, Guazzelli É (2004). Inertial migration of rigid spherical particles in Poiseuille flow. J Fluid Mech.

[R48] Oberdorster G (2000). Toxicology of ultrafine particles, In vivo studies, Philos. T R Soc A.

[R49] Pal AK, Bello D, Budhlall B, Rogers E, Milton DK (2012). Screening for Oxidative Stress Elicited by Engineered Nanomaterials: Evaluation of Acellular DCFH Assay. Dose-Response.

[R50] Pan M, Eiguren-Fernandez A, Hsieh H, Afshar-Mohajer N, Hering SV, Lednicky J, Fan ZH, Wu CY (2016). Efficient collection of viable virus aerosol through laminar-flow, water-based condensational particle growth. J Appl Microbiol.

[R51] Sameenoi Y, Koehler K, Shapiro J, Boonsong K, Sun Y, Collett J, Volckens J, Henry CS (2012). Microfluidic Electrochemical Sensor for On-Line Monitoring of Aerosol Oxidative Activity. J Am Chem Soc.

[R52] Sauvain J-J, Deslarzes S, Storti F, Riediker M (2015). Oxidative Potential of Particles in Different Occupational Environments: A Pilot Study. Ann Occup Hyg.

[R53] Shinyashiki M, Eiguren-Fernandez A, Schmitz DA, Di Stefano E, Li N, Linak WP, Cho S-H, Froines JR, Cho AK (2009). Electrophilic and redox properties of diesel exhaust particles. Environ Res.

[R54] Singh U, Jialal I (2006). Oxidative stress and atherosclerosis. Pathophysiology.

[R55] Steenhof M, Gosens I, Strak M, Godri K, Hoek G, Cassee F, Mudway I, Kelly F, Harrison R, Lebret E, Brunekreef B, Janssen N, Pieters R (2011). In vitro toxicity of particulate matter (PM) collected at different sites in the Netherlands is associated with PM composition, size fraction and oxidative potential –the RAPTES project. Particle and Fibre Toxicology.

[R56] Stolzenburg M, Kreisberg N, Hering S (1998). Atmospheric size distributions measured by differential mobility optical particle size spectrometry. Aerosol Sci Technol.

[R57] Swanson KJ, Kado NY, Funk WE, Pleil JD, Madden MC, Ghio AJ (2009). Release of the Pro-Inflammatory Markers by BEAS-2B Cells Following In Vitro Exposure to Biodiesel Extracts. The Open Toxicology Journal.

[R58] Uttara B, Singh AV, Zamboni P, Mahajan RT (2009). Oxidative Stress and Neurodegenerative Diseases: A Review of Upstream and Downstream Antioxidant Therapeutic Options. Curr Neuropharmacol.

[R59] Uzu G, Sauvain J-J, Baeza-Squiban A, Riediker M, Sánchez Sandoval Hohl M, Val S, Tack K, Denys S, Pradère P, Dumat C (2011). In vitro Assessment of the Pulmonary Toxicity and Gastric Availability of Lead-Rich Particles from a Lead Recycling Plant. Environ Sci Technol.

[R60] Valko M, Morris H, Cronin MTD (2005). Metals, toxicity and oxidative stress. Curr Med Chem.

[R61] Verma V, Ning Z, Cho AK, Schauer JJ, Shafer MM, Sioutas C (2009). Redox activity of urban quasi-ultrafine particles from primary and secondary sources. Atmos Environ.

[R62] Verma V, Rico-Martinez R, Kotra N, King L, Liu J, Snell TW, Weber RJ (2012). Contribution of water-soluble and insoluble components and their hydrophobic/hydrophilic sub-fractions to the reactive oxygen species-generating potential of fine ambient aerosols. Environ Sci Technol.

[R63] Wang D, Pakbin P, Shafer MM, Antkiewicz D, Schauer JJ, Sioutas C (2013). Macrophage reactive oxygen species activity of water-soluble and water-insoluble fractions of ambient coarse, PM2.5 and ultrafine particulate matter (PM) in Los Angeles. Atmos Environ.

[R64] Wang J, Asbach C, Fissan H, Huelser T, Kuhlbusch T, Thompson D, Pui D (2011). How can nanobiotechnology oversight advance science and industry: Examples from environmental, health and safety studies of nanoparticles (nan-EHS). J Nanopart Res.

[R65] Wessels A, Birmili W, Albrecht C, Hellack B, Jermann E, Wick G, Harrison RM, Schins RPF (2010). Oxidant Generation and Toxicity of Size-Fractionated Ambient Particles in Human Lung Epithelial Cells. Environ Sci Technol.

[R66] Xia T, Kovochich M, Nel A (2006). The role of reactive oxygen species and oxidative stress in mediating particulate matter injury. Occup Environ Med.

[R67] Yang A, Janssen NAH, Brunekreef B, Cassee FR, Hoek G, Gehring U (2016). Children’s respiratory health and oxidative potential of PM2.5: the PIAMA birth cohort study. Occup Environ Med.

